# Posterior reversible encephalopathy syndrome post stem cell transplantation in sickle cell disease: case series and literature review

**DOI:** 10.3389/fmed.2024.1330688

**Published:** 2024-05-01

**Authors:** Hussain A. BinAmir, Ali AlAhmari, AlWaleed AlQahtani, Gamal Mohamed, Fawaz Alotaibi, Mohamed AlShamrani, Ali AlSaeed, Suwaidi AlGhanmi, Alaa Heji, Abdulrahman Alreshaid, Ammar AlKawi, Adel AlHazzani, Mohamed AlZawahmah, Ashfaq Shuaib, Fahad Al-Ajlan, Fahad AlMohareb

**Affiliations:** ^1^Neuroscience Center, King Faisal Specialist Hospital and Research Centre, Riyadh, Saudi Arabia; ^2^Hematology, Stem Cell Transplant and Cellular Therapy Department, King Faisal Specialist Hospital and Research Centre, Riyadh, Saudi Arabia; ^3^Radiology Department, King Faisal Specialist Hospital and Research Centre, Riyadh, Saudi Arabia; ^4^Department of Biostatistics, Epidemiology, and Scientific Computing, King Faisal Specialist Hospital and Research Centre, Riyadh, Saudi Arabia; ^5^Department of Medicine, AlFaisal University, Riyadh, Saudi Arabia; ^6^Department of Medicine University of Alberta, Edmonton, AB, Canada

**Keywords:** posterior reversible encephalopathy syndrome, stem cell transplantation, sickle cell disease, bi-hemispheric infarctions, cerebral microbleeds

## Abstract

**Introduction:**

Posterior reversible encephalopathy syndrome (PRES) is a serious neurological syndrome that may develop following immunosuppressive therapy for stem cell transplantation (SCT). We report 8 patients with sickle cell disease (SCD) who developed PRES, which is likely to be related to immunosuppression.

**Methods:**

This is retrospective cohort analysis of the SCD registry at the King Faisal Specialist Hospital and Research Center (KFSHRC) in Riyadh, Saudi Arabia. Inclusion criteria included all adults SCD patients who underwent SCT from 2011 until 2022. We explored all cases of PRES in patients with SCT. PRES was diagnosed with MRI imaging showing reversible vasogenic cerebral edema associated with neurological symptoms including severe headache, seizures, encephalopathy, delirium, and visual disturbances.

**Results:**

During ten years follow-up (2011–2022) we found 8 patients with PRES (age range between 14 to 37 years at diagnosis) PRES occurred 8 to 124 days following SCT in 7 cases and one patient developed PRES 8 months prior to SCT. All patients were on immunosuppressive medications, including tacrolimus, cyclosporine, sirolimus and or mycophenolate mofetil. Headache, seizures, visual hallucinations, confusion, and drowsiness were the most common presenting symptoms. MRI showed abnormalities in the occipital, parietal and frontal lobes in most cases. Recovery was complete in all patients and no recurrences were noted. Two patients had graft versus host disease (GVHD). We compared risk factors for PRES among the 8 cases and 136 SCT in SCD patients who did not develop PRES. There was a significant association between PRES and imaging abnormalities, including previous bi-hemispheric infarctions (*p* = 0.001), and cerebral microbleeds (CBMs). PRES was strongly associated with presence (*p* = 0.006), size (*p* = 0.016) and number (*p* = 0.005) of CMBs.

**Conclusion:**

PRES can develop days to weeks following SCT in patients with SCD, and is associated with immunosuppressive therapy, previous bi-hemispheric infarctions and CMB. Prompt recognition and intervention leads to good recovery.

## Introduction

Posterior reversible encephalopathy syndrome (PRES) refers to the development of reversible vasogenic cerebral edema leading to neurological symptoms including severe headache, seizures, encephalopathy, delirium, and visual disturbances (1). Common underlying etiologies that may precipitate PRES include sudden increase in blood pressure, autoimmune disorders and medications, especially cytotoxic drugs and eclampsia (2). Diagnosis is usually confirmed with brain imaging, particularly MRI which shows characteristic changes suggestive of vasogenic edema, especially in the occipital, temporal and parietal regions (2). While the underlying mechanisms are not fully understood, inflammation and endothelial cell dysfunction resulting in increased leakage of the blood brain barrier (BBB) are important contributing factors (3).

PRES has been reported with several hematological disorders including thalassemia and sickle cell disease (SCD) (4–6). In SCD, most cases have been reported in children in close proximity to stem cell transplantation (SCT). PRES in adult SCD patients is rare (7–11). To our knowledge, there are only two reports of PRES in SCD in proximity to SCT (7, 8). We report 8 SCD cases from our institution who underwent SCT and developed PRES.

## Methods

We reviewed the Stem Cell Transplantation database at the King Faisal Specialist Hospital and Research Center (KFSHRC) in Riyadh, Kingdom of Saudi Arabia (KSA) for all patients who underwent stem cell transplantation for SCD between 2011 and 2022. This database includes all SCD patients indicated for SCT, indications of SCT include: SCD with CNS events (stroke, TIA, CNS vasculopathy), SCD with more than two acute chest syndromes in last 2 years, more than two joints with avascular necrosis, more than 2 vaso-occlusive crisis per year for past 2 years, sickle cell nephropathy, recurrent priapism.

We studied the SCD transplant database for potential risk factors for PRES in SCT treated patients. These variables include demographic data, clinical features, laboratory investigation, previous radiological abnormalities and medications. We also evaluated the presence of any acute illness, hypertensive crises, medications changes and the proximity to the timing of conditioning and the development of PRES. The diagnosis of PRES is made with brain MRI showing reversible vasogenic edema in bilateral cortical or/and subcortical areas. We used chi-square and Fisher’s exact tests for association between demographics, clinical, laboratory and radiological variables and PRES.

## Results and summary

There were 144 patients who underwent SCT for SCD. PRES developed in 8 patients. The details of their clinical presentations and laboratory features are shown in Tables 1, 2. There was no difference in the age at the time of SCT, hematological or clinical parameters between the two groups. While there was no significant difference in the long-term blood pressures between the two groups, 7 of 8 patients were hypertensive during the acute PRES illness with systolic blood pressure between 140 and 170 mmHg. The time between the SCT and development of PRES varied from 8 days to 4 months after the transplant. The time interval between SCT and PRES was less than 40 days in 5 of 7 cases.

**Table 1:** Clinical, hematological and treatment variables in patients with and without PRES.

Variable	PRES (*n* = 8)	Non-PRES (*n* = 136)	*p* value
Average age at transplant	21	22	0.109
Average blood pressure — mm Hg	110/68	108/65	0.537
Prior history of stroke (%)	1 (12.5)	15 (11)	0.898
Vaso-occlusive crisis (%)	7 (87.5)	118 (86.7)	0.952
Acute chest syndrome (%)	3 (37.5)	39 (28.6)	0.594
**Hematological features**			
WBC — 10^9/L	7	8.8	0.275
Hb — g/dL	93	93	0.142
HBS — %	45	52	0.960
Reticulocyte — %	6.6	8.4	0.578
LDH — U/L	281	305	0.558
G6PD quantitative — U/g Hgb	16	15	0.539
**Medications**			
Antiplatelet-Aspirin (%)	1 (12.5)	6 (4.4)	0.452
**Previous radiological abnormalities**	**7 out of 8 had had MRI done** (%)	**101 out of 136 have had MRI done** (%)	
Cortical infarction	1 (14.2)	13 (12.8)	0.914
Subcortical infarction	3 (42.8)	16 (15.8)	0.169
Lacune	1 (14.2)	14 (13.8)	0.975
Right hemisphere	2 (28.5)	2 (1.9)	0.001
Left hemisphere	0	6 (5.9)	
Bi-hemispheric infarction	2 (28.5)	10 (9.9)	
CMB: presence	2 (28.5)	4 (3.9)	0.006
CMB number			
CMB 0	5 (71.4)	97 (96)	0.016
CMB 1	0	1 (0.99)	
CMB 2–4	1 (14.2)	2 (1.9)	
CMB > 5	1 (14.2)	1 (0.99)	
CMB size			0.005
CMB < 5 mm	0	1 (0.99)	
CMB > 5 mm	2 (28.5)	2 (1.9)	
WMD	2 (28.5)	27 (26.7)	0.915

WBC: white blood count; Hb: hemoglobin; HBS: hemoglobin S; LDH: lactate dehydrogenase; G6PD: glucose-6-phosphate dehydrogenase; Cerebral microbleed CMB, white matter disease: WMD.

**Table 2:** Clinical details, medications and radiological features of the patients presenting with PRES.

Case	Age/sex	Age at Tx	Days from Tx	BP at onset	GVHD prophylaxis/GVHD type	Conditioning	Clinical and MRI findings acutely	Clinical and radiological follow up
1	20/M	15	12 prior to Tx	110/68	CSA No GVHD	CY/FLU/ATG	Seizure. Bilateral FLAIR cortical and subcortical occipital, temporal, parietal as well as frontal lobes hyperintensities.	No focal neurological deficits nor seizure, stopped ASM after 2 years. Full resolution of PRES findings
2	26/F	19	8	179/93	CSA Skin GVHD grade 3, Gut GVHD grade 4.	Bu/FLU/ATG	Altered mental status, visual loss and seizure. Bilateral FLAIR cortical and subcortical occipital, basal ganglia, capsular region hyperintensities with microhemorrhages.	No focal neurological deficits and not maintained on ASMs Partial resolution of abnormal high signal intensities. Evolution of hemorrhage within bilateral occipitoparietal region.
3	28/F	23	22	156/103	CSA No GVHD	Bu/FLU/ATG	Headache Bilateral FLAIR cortical and subcortical occipital-parietal hyperintensity, small foci of cortical diffusion restriction in left parietal lobe, right centrum semiovale FLAIR hyperintensity.	No focal neurological deficits. Few foci of high T2/FLAIR signal in the right centrum semiovale and subcortical white matter.
4	21/F	17	8	156/95	CSA No GVHD	Bu/FLU/ATG	Headache and seizure. Parieto-occipital subcortical hyperintensity.	No focal neurological deficits, no ASM. Bilateral occipital high signal suggestive of PRES.
5	21/M	17	124	141/87	MMF CSA No GVHD	Bu/FLU/ATG	Visual hallucinations, confusion, seizure. Bilateral symmetrical insular cortex, cingulate gyrus hyperintensity, patchy right frontal and left parietotemporal cortical hyperintensity as well as in the left dorsal thalamus. There is associated restricted diffusion. There are multiple FLAIR hyperintensity without restricted diffusion in the right parietooccipital region. Central pons hyperintensity. There is also hippocampus hyperintensity with mild diffusion restriction.	No focal neurological deficits and not maintained on ASMs. Interval improvement of previously described areas of diffusion restriction and abnormal high T2/FLAIR signal intensity with residual minimal diffusion restriction.
6	17/F	15	62	160/100	TAC SRL MMF Gut GVHD grade 4.	rATG/Thio/FLU/TBI200	Blurred vision, headache and seizure. Cortical and subcortical FLAIR hyperintensity in bilateral, frontal, parietal, occipital parasagittal regions with associated restricted diffusion in the right posterior frontal and left occipital region. There is leptomeningeal enhancement corresponding to the hyperintense areas (likely reactive).	No focal neurological deficits and not maintained on ASMs. Partial resolution of PRES findings
7	40/F	38	28	156/87	TAC No GVHD	Bu/FLU/ATG	Agitation and confusion. Periventricular white matter hyperintensity.	No focal neurological. Full resolution of PRES findings
8	19/F	18	37	140/91	SRL MMF No GVHD	rATG/Thio/FLU/CY/TBI	Agitation, confusion and seizure. Multiple patchy bilateral hyperintensity in periventricular white matter, corona radiata, centrum semiovale, head of caudate.	No focal neurological deficits but still maintained on levetiracetam 500 mg twice a day for post stroke-epilepsy. No FU MRI

Tx: transplant; BP: blood pressure; M: male, F: female; GVHD: Graft versus host disease; TAC: tacrolimus, SLM: sirolimus, CSA: cyclosporine, MMF: mycophenolate mofetil; Bu: busulfan, Flu: fludarabine, ATG: antithymocyte globulin, Cy: Cyclophosphamide, TBI: total body irradiation, ASM: antiseizure medication.

The most common symptoms associated with PRES at the time of admission included sudden confusion, headache, visual symptoms, seizures, agitation and drowsiness. As shown in Table 2, most patients had mild disease, defined as cortical and/or subcortical edema without hemorrhage, mass effect or herniation (12), and all made full recovery. No patients developed recurrent PRES in follow-up ranging from 1 to 7 years.

Details of GVHD prophylaxis and conditioning regimen are shown in Table 2. Tacrolimus, sirolimus, mycophenolate and cyclosporine were the most used medications for GVHD prophylaxis. The conditioning medications are also shown in Table 2 and included busulfan (Bu)/fludarabine (Flu), antithymocyte globulin (ATG) or Flu/Cyclophosphamide (Cy)/ATG in most patients.

**Figure 1:**
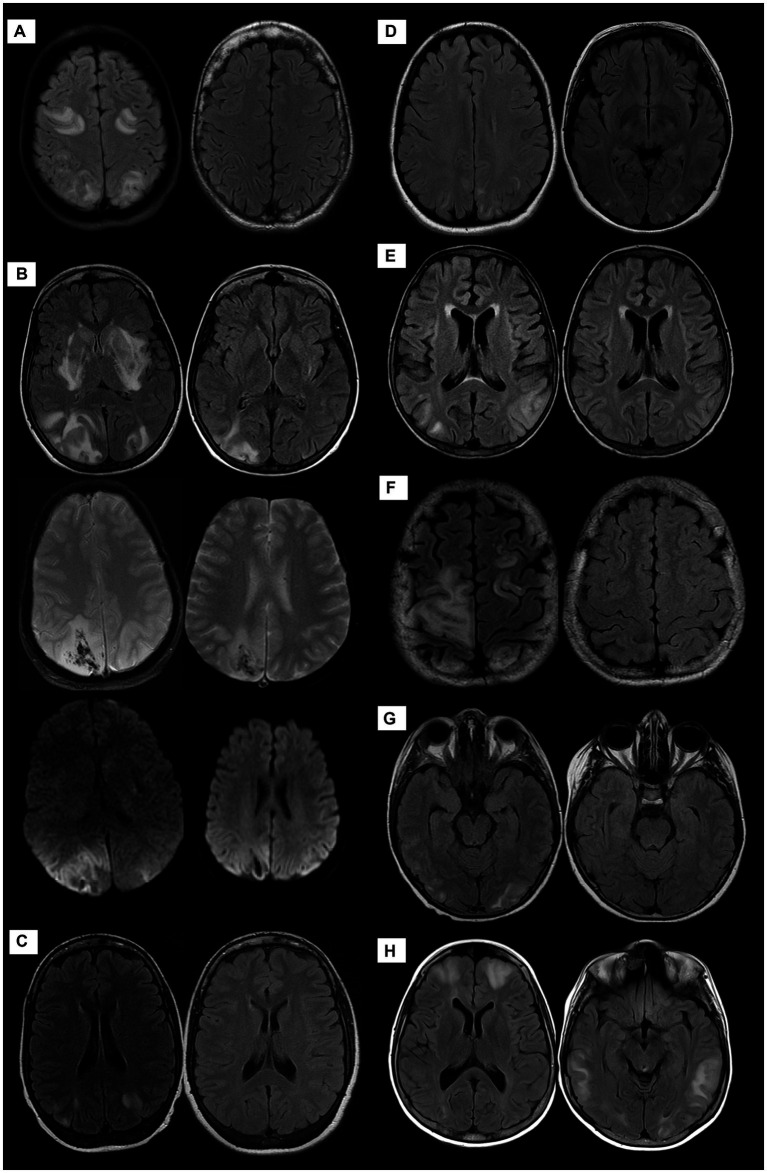
MRI of the brain of all patients demonstrating PRES manifestations at variable degrees; patient 1 **(A)**: the initial exam of AXIAL FLAIR weighted images-(WI)-left side- shows predominantly sub-cortical signal alteration involving bilateral fronto-parietal and occipital lobes with complete resolution on the follow-up study right side. Patient 2 **(B)** Cortical and sub-cortical diffuse signal alteration at the bilateral parieto-occipital lobes with diffusion restriction and hemorrhage evident by blooming artifacts on SWI as well as unusual signal alteration at bilateral basal ganglia. Follow-up images at right side exhibit significant improvement with residual signal alteration of hemorrhagic PRES. Patient 3 **(C)**, Patient 4 **(D)**, and Patient 5 **(E)**: AXIAL FLAIR WI demonstrate predominantly sub-cortical signal alteration involving bilateral parieto-occipital lobes with complete resolution on follow-up images right side except for patient 4, no follow-up images. Patient 6 **(F)**, Patient 7 **(G)**, and Patient 8 **(H)** AXIAL FLAIR WIs demonstrate predominantly sub-cortical white matter signal alteration at different distributions, mainly parieto-occipital to a lesser extent involving fronto-temporal lobes with complete resolution on follow-up images right side except for patient 8, no follow-up images.

The imaging findings are shown in Figure 1. The diagnosis of PRES was confirmed on MRI imaging in most patients. The hyperintensities on FLAIR images were most frequently seen bilaterally cortically and sub-cortically in the occipital, parietal and temporal lobes. No patients developed ICH or SAH.

A significant association was observed between imaging abnormalities and PRES. The presence of bi-hemispheric infarctions (*p* = 0.001), presence (*p* = 0.006), size (*p* = 0.016) and number (*p* = 0.005) of cerebral-microbleed (CMB) were higher risk of PRES. There was no significant association between the presence or severity of white matter hyperintensities [as measured by Fazekas score (13)] and the risk of PRES.

## Discussion

We report eight patients with SCD who developed PRES after SCT, four of whom are adults. The time between the SCT and development of PRES varied from 8 days to 4 months after transplant. The time to PRES was less than 40 days from SCT in 5 of 7 cases and 8 months prior to SCT in one patient. We did not identify any preceding precipitating factors, and the clinical course was benign with no recurrences in our patients. We noted that the presence of previous bi-hemispheric cerebral infarction and CMBs increased the risk of PRES. The presence of white matter hyperintensities did not appear to be associated with increased risk.

Although rare in adults, PRES has been frequently reported in children with SCD. Chronic hemolysis can lead to the release of erythrocyte damage-associated molecular protein molecules (cDAMPs). In addition, Toll-like receptor 4 promotes the release of oxygen reactive species. These together with neutrophil extracellular traps, can result in endothelial cell injury and injury to the blood–brain barrier (8). Conditioning chemotherapy can also result in endothelial injury and increase the risk of PRES in children (8, 14). Endothelial cell dysfunction is also noted after HSCT, similarly, is driven by the predominant presence of pro-inflammatory factors such as complement activation, cDAMPs, and Toll-like receptors (15). Complement activation is not known if it has a role in the pathophysiology of the endothelial dysfunction in PRES, compared to post-HSCT. Genetic abnormalities in the complement pathway have been associated with some thrombotic microangiopathy syndromes, for instance transplant-associated thrombotic microangiopathy (16). Furthermore, thrombotic microangiopathy can develop with or without SCT in patients with SCD and has been successfully treated with eculizumab to improve symptoms of PRES (8).

SCT and preconditioning are frequently associated with neurotoxicity including PRES (14). The combination of preexisting endothelial injury from chronic on-going hemolysis and the additional injury from cytotoxic/immunosuppressive medications can place patients with SCD at a greater risk of thrombotic thrombocytopenia, veno-occlusive disease and PRES (17, 18). In children with SCD with SCT, PRES has been reported in up to 22% of patients in one large study from Rome (19). The incidence of PRES seems to be significantly less frequent in adult SCD patients and SCT, with only two previous cases prior to our report (7, 8). The reasons for such age-related discrepancy are not yet clear. We cannot exclude the possibility that this may be related to under-reporting of cases in adults.

In children with hemoglobinopathies, PRES has been reported in patients treated with calcineurin inhibitors (cyclosporin, sirolimus and tacrolimus) (4, 6, 19, 20). In one series of children with SCD and thalassemia who underwent SCT from Rome (age range 2–17 years old), PRES was reported in 31 of 222 cases. All patients had been treated with cyclosporine or tacrolimus and PRES developed in most patients within 100 days from SCT (19). In another series of 313 consecutive SCT in children from Bologna, PRES was reported in 26 (8.3%) patients and occurred in higher frequency in patients with hemoglobinopathies (20). The time from SCT to PRES ranged from 5 to 352 days. The authors noted an association between fludarabine-based conditioning and the risk of PRES in these children.

As shown in Table 3, there are rare reports of PRES in adult patients with SCD (7–11). To our knowledge, there are only two previous cases of SCD in which SCT was complicated by PRES. Sweany et al. reported 3 patients with recurrent PRES in patients with SCD, systemic lupus erythematosus and acute myeloid leukemia. All patients had evidence of endothelial cell injury (schistocyte formation and increased LDH). PRES was associated with SCT in one of the three cases. The time interval between SCT and PRES was not mentioned (7). Bhunia et al. reported one pediatric and one adult patient with SCD who developed PRES following SCT (8). Interestingly, the authors considered transplant-associated thrombotic microangiopathy as predisposing to PRES and treatment with eculizumab seems to result in quick recovery (8). Recurrent episodes of PRES in SCD have also been reported by Nair and Testai. The patient did not undergo SCT (10). Throughout eight years of follow-up, we did not observe any recurrent episodes of PRES in our patients.

**Table 3:** Literature review: adult patients with PRES in relation to SCD with or without transplant.

Study	Nr of cases	Age/sex	Diagnosis/transplant	Median onset of Sx	Risk factors (RF)	Presentation	MRI	Clinical and radiological follow up
Bhunia et al. (8)	2	25 and 8	SCD/Tx	18- and 74-days post Tx	Ongoing hemolysis and TATM	Headache, HTN, seizure, altered level of consciousness.	Vasogenic edema in the cortical and subcortical zones of the cerebellar and cerebral hemispheres, pons, thalami, and posterior limbs of internal capsule. Left sided intracranial hemorrhage parietal cortex and subcortical white matter.	Resolved seizures on 2 ASM, residual neurocognitive defect. No FU MRI done. Improved neurological status, no neurocognitive decline, no FU MRI.
Vargas and Testai (9)	4	25, 34, 30, and 32.	SCD	-	HTN, TAC	HTN, seizure. HTN, seizures and visual blur. Seizure and normotensive. HTN, altered mental status, seizure	2 cases had FLAIR parietooccipital hyperintensities. Third case: asymmetric FLAIR cortical and subcortical frontoparietal and periventricular hyperintensities. Fourth case: FLAIR occipital cortical and subcortical hyperintensities.	Improved visual Sx. Improved MRI.
Geevasinga et al. (11)	2	17 years old Thal 30 years SCD post Tx	Thal and SCD/Tx	36-days after transplant	Unrecognizable RF for pt. 1 (Thal). CSA for pt. 2 (SCD).	Altered mental status, right side weakness, seizures and HTN. HTN, vison loss and severe headache.	Bilateral FLAIR cortical and subcortical hyperintensities, brainstem and cerebellum. Bilateral occipital infarctions.	Thal patient passed away of sepsis. NO MRI FU. SCD patient passed away of GVHD. NO MRI FU.
Nair and Testai et al. (10)	1	First episode at 27 years old and the second episode at age 32.	SCD	-	Sickle cell crisis.	New onset seizure. Headache and encephalopathy. Normotensive.	MRI at first event: FLAIR cortical and subcortical occipital, parietal, frontal hyperintensities. MRI at second event: FLAIR cortical and subcortical parieto-occipital hyperintensities.	Improved after first event. Recur after 4 years. Improved after second event.
Sweany et al. (7)	1	Recurrent PRES at age of 56	SCD	–	Sickle cell crisis, respiratory failure.	HTN, encephalopathy and seizure. Headache and blurred vision.	MRI at first event: Vasogenic edema of cortical and subcortical occipital lobe. MRI at second event: Vasogenic edema of frontal, parietal and occipital lobes.	Improved mental status and MRI. Recur after 4 months. Improved MRI after 1 month.

SCD: sickle cell disease; Tx: transplant; Thal: thalassemia; TAC: tacrolimus; CSA: cyclosporine; TATM: transplant associated thrombotic microangiopathy; ASM: anti-seizure medications; FU: follow-up.

Differentiation of PRES from ischemic or hemorrhagic stroke is important and can sometimes be challenging. Sickle cell disease patients are at an increased risk of stroke and moyamoya disease (21). The clinical presentation is mostly with focal neurological symptoms; and the imaging, especially MRI-DWI shows characteristic findings that are helpful in making the correct diagnosis (1, 2). Early diagnosis, especially in patients with ischemic stroke, is important as the patients can be candidates for reperfusion treatment with intravenous alteplase or endovascular clot removal. Patients with PRES may require rapid blood pressure reduction, which can sometimes be harmful in acute ischemic stroke.

There are strengths to our study. This is a comprehensive review from an academic center with experience and a large volume of stem cell transplantation for SCD. The patients were evaluated by hematologists and neurologists and had extensive investigations to determine the etiology. The follow-up is comprehensive and clinical course was compared to a large number of SCD transplanted patients who did not develop PRES.

However, there are limitations to the study. On the one hand, this is a single center study which reflects the experience from the Kingdom of Saudi Arabia and therefore the results cannot be generalized. On the other hand, the data in the transplant registry was collected prospectively, our study is a retrospective analysis and carries the risk of inadequate data collection and evaluation. Future research is warranted to include more patients with collaboration with other transplant institutes to test the clinical implications of findings noted on MRI as predictor of PRES in SCD patients and for which any intervention for endothelial dysfunction could play a role.

In summary, we present four adult SCD patients post SCT in addition to two prior reported cases. While most patients were hypertensive, we did not identify an acute hypertensive or sickle cell crisis in any patient preceding PRES. Immunosuppressive treatment or conditioning may have been contributing factors in some patients. MRI can be used as a probable predictive tool of PRES in patients with SCD undergoing SCT. The clinical course was mild in most patients and none experienced recurrence.

## Data availability statement

The raw data supporting the conclusions of this article will be made available by the authors, without undue reservation.

## Ethics statement

The studies involving humans were approved by the Research Ethics Committee (REC) of King Faisal Specialist Hospital and Research Centre, Riyadh, Saudi Arabia. The studies were conducted in accordance with the local legislation and institutional requirements. Written informed consent for participation was not required from the participants or the participants' legal guardians/next of kin in accordance with the national legislation and institutional requirements. Written informed consent was obtained from the individual(s) for the publication of any potentially identifiable images or data included in this article.

## Author contributions

HB: Conceptualization, Data curation, Formal analysis, Investigation, Methodology, Writing – original draft, Writing – review & editing. AAlA: Conceptualization, Data curation, Formal analysis, Methodology, Validation, Writing – review & editing. AAlQ: Conceptualization, Data curation, Methodology, Visualization, Writing – review & editing. GM: Data curation, Formal analysis, Writing – review & editing. FAlo: Conceptualization, Data curation, Formal analysis, Writing – review & editing. MAlS: Conceptualization, Data curation, Formal analysis, Writing – review & editing. AAlS: Conceptualization, Data curation, Formal analysis, Writing – review & editing. SA: Conceptualization, Data curation, Formal analysis, Writing – review & editing. AH: Conceptualization, Data curation, Formal analysis, Writing – review & editing. AAlr: Conceptualization, Data curation, Writing – review & editing. AAlK: Conceptualization, Data curation, Writing – review & editing. AAlH: Conceptualization, Data curation, Writing – review & editing. MAlZ: Conceptualization, Data curation, Writing – review & editing. AS: Conceptualization, Data curation, Formal analysis, Methodology, Supervision, Writing – original draft, Writing – review & editing. FA-A: Conceptualization, Data curation, Methodology, Supervision, Validation, Writing – review & editing. FAlM: Conceptualization, Data curation, Writing – review & editing.
